# Extension of Term

**Published:** 1884-08

**Authors:** 


### Extension of Term.

At a recent meeting of the Regents of the University of Michigan, the term of the college, which has hitherto been six months, was extended to nine months. The desire has existed for some time to have this extension; it has become important for various considerations. It is desirable, on the part of the authorities, to have all the departments continued for the same time. It has also been found that the curriculum in the dental college is greater than can be satisfactorily mastered in the time assigned to it. Even those who have devoted three six-months terms have often expressed themselves as desirous of having more time, and very few have gone through in two courses without expressing regret at the shortness of the time. Certain it is that this extension will be acceptable to the profession, and to the students who enter upon their course in this Institution. Two nine-months courses will in all cases be required for graduation. By this extension, students will have more time for work and study, and will not be pressed as heretofore. The aim is to make thorough work, and for this, time and facilities must be afforded.

